# BraMARS: An Interpretable Histopathology‐Driven Deep Learning Model for Brain Metastasis Risk Stratification in Surgically Resected Limited‐Stage SCLC

**DOI:** 10.1002/advs.77075

**Published:** 2026-08-14

**Authors:** Zijian Yang, Taolue Wang, Shilong Liu, Zicheng Zhang, Yibo Zhang, Fan Yang, Bo Yu, Shuaishuai Gao, Yu Chen, Lin Yang, Meng Zhou

**Affiliations:** ^1^ Institute of Genomic Medicine School of Biomedical Engineering Wenzhou Medical University Wenzhou People's Republic of China; ^2^ Department of Thoracic Radiation Oncology Harbin Medical University Cancer Hospital Harbin People's Republic of China; ^3^ Department of Pathology National Cancer Center/National Clinical Research Center for Cancer/Cancer Hospital Chinese Academy of Medical Sciences and Peking Union Medical College Beijing People's Republic of China; ^4^ AMG Nephrology Avera McKennan Hospital Sioux Falls South Dakota USA

**Keywords:** brain metastasis, deep learning, prophylactic cranial irradiation, small cell lung cancer

## Abstract

Brain metastasis (BM) is a major cause of mortality in limited‐stage small‐cell lung cancer (LS‐SCLC). Prophylactic cranial irradiation (PCI) reduces BM incidence but carries neurotoxicity and lacks individualized risk assessment. Here, we developed BraMARS, an explainable deep learning model that estimates future BM risk from routine H&E‐stained whole‐slide images of resected LS‐SCLC. BraMARS demonstrates robust discriminatory performance across independent cohorts, with AUCs ranging from 0.738 to 0.944, and stratifies patients into high‐risk and low‐risk groups with significantly different disease‐free survival, overall survival, and brain metastasis‐free survival. Retrospective simulation shows BraMARS‐guided risk stratification could reduce PCI exposure in 19.3% of low‐risk predicted patients while improving identification of high‐risk‐predicted patients by 84.4%. Histopathologic attribution and proteomic analyses linked higher scores to distinct tissue patterns and programs involving mitochondrial metabolism, reactive‐oxygen‐species detoxification, and DNA repair. Overall, BraMARS provides a biologically interpretable histopathology‐based framework for estimating subsequent BM risk in resected LS‐SCLC, with potential to support individualized intracranial risk assessment, intensified MRI surveillance, and hypothesis generation for prospective BM‐prevention strategies.

## Graphical abstract

This study presents BraMARS, an explainable deep learning model that estimates future brain metastasis risk in surgically resected limited‐stage small‐cell lung cancer using routine H&E‐stained whole‐slide images. By linking model‐attributed spatial histopathology with clinical outcomes and proteomic programs, BraMARS provides a biologically interpretable framework for individualized intracranial risk assessment, with potential to inform prophylactic cranial irradiation discussions, MRI surveillance strategies, and future brain metastasis prevention studies.

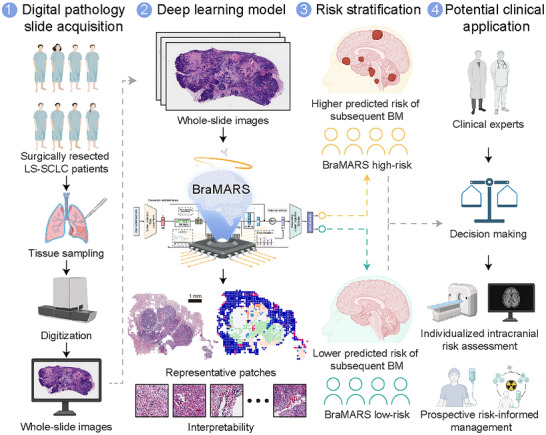

## 1 Introduction

Small cell lung cancer (SCLC) is a highly aggressive neuroendocrine malignancy, accounting for approximately 15% of all lung cancer cases [1]. SCLC is characterized by rapid growth, early metastasis, and a poor prognosis [2]. Despite a high initial response rate to platinum‐based chemotherapy and radiotherapy, disease relapse is common, and the brain represents one of the most clinically consequential sites of metastasis [3]. Brain metastasis (BM) significantly worsens survival and leads to cognitive decline and functional impairment. BMs are frequently diagnosed at relapse, when therapeutic options are limited and outcomes are poor [4, 5]. Therefore, effective prevention and early risk stratification of BM remain critical for improving long‐term management in SCLC.

Prophylactic cranial irradiation (PCI) has historically been used as an intracranial preventive strategy for patients with limited‐stage SCLC (LS‐SCLC) who respond to initial therapy. Evidence from meta‐analyses indicates that PCI reduces the cumulative incidence of BMs from approximately 58.6% to 33.3% with a corresponding improvement in three‐year survival from 15.3% to 20.7% [6]. Another pooled analysis of 28 retrospective studies demonstrated a significant beneficial effect of PCI vs. no PCI with a median survival of 27.8 months in the PCI group vs. 18.8 months in the no‐PCI group [7]. However, PCI is also associated with clinically relevant toxicities, including cognitive impairment and fatigue, which complicate its benefit–risk profile. In the modern MRI surveillance era, the role of PCI has become increasingly individualized, particularly given concerns regarding neurotoxicity, heterogeneity in baseline BM risk, and evolving surveillance strategies. PCI selection is still largely based on clinicopathological factors and physician–patient discussion rather than individualized prediction of future BM risk [8, 9, 10, 11]. Current imaging modalities, such as magnetic resonance imaging (MRI) and computed tomography (CT), are primarily used for detecting established intracranial disease rather than to proactively estimate future BM risk [12]. Therefore, more accurate and clinically practical approaches are needed to stratify BM risk and inform individualized PCI risk discussion in LS‐SCLC.

In this study, we address this gap by developing and validating BraMARS (Brain Metastasis Assessment and Risk Stratification), a histopathology‐based explainable risk stratification model designed to estimate future BM risk from routinely acquired H&E‐stained whole‐slide images in patients with surgically resected LS‐SCLC. Rather than serving as a definitive PCI decision tool, BraMARS was intended to provide a biologically interpretable framework for identifying patients with elevated BM risk who may warrant individualized discussion of PCI, intensified MRI surveillance, or other intracranial preventive strategies, while potentially reducing treatment exposure among patients predicted to be at relatively lower BM risk. Furthermore, we performed integrative multi‐modal analysis combining spatially resolved histopathological patterns with proteomic profiling to explore the biological basis of BraMARS risk stratification.

## 2 Results

### 2.1 Cohort Characteristics and Study Design

**FIGURE 1:**
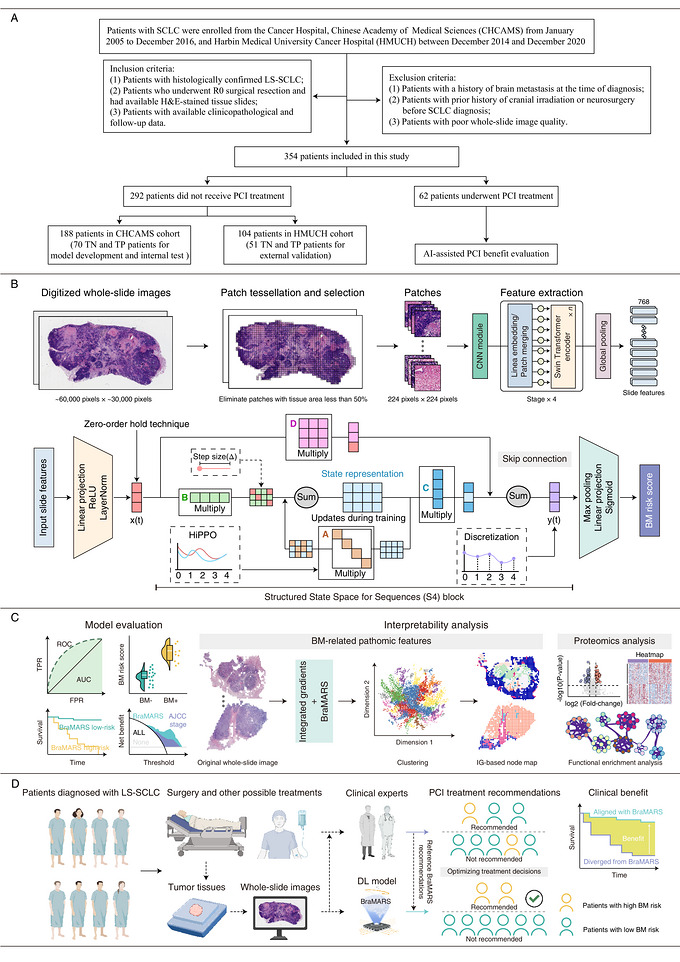
Study overview and BraMARS deep learning architecture. (A) Flow diagram illustrating patient enrollment from two independent medical centers (CHCAMS and HMUCH), inclusion/exclusion criteria, and cohort allocation for model development, validation, and clinical applicability assessment. (B) Schematic representation of the BraMARS deep learning model architecture consisting of three main components: WSI preprocessing and patch‐level feature extraction, sequential feature encoding using S4 and MIL aggregation for final prediction. (C) Workflow of model evaluation and interpretability. (D) Conceptual workflow illustrating how BraMARS may be incorporated into future prospective studies to support intracranial‐risk stratification, individualized PCI discussion, and MRI‐surveillance planning. TP, true positive; TN, true negative; WSI, whole‐slide image; S4, Structured State Space for Sequences; MIL, multiple instance learning.

We conducted a retrospective, multicenter study comprising 354 patients with resected LS‐SCLC, including 245 patients from the CHCAMS cohort and 109 from the HMUCH cohort (Figure 1). Baseline demographic and clinical characteristics are summarized in Table 1. The mean age was 56.57 ± 10.06 years in the CHCAMS cohort and 59.26 ± 7.81 years in the HMUCH cohort, with a predominance of male patients in both cohorts. Smoking history was prevalent in both cohorts, accounting for 64.49% of patients in CHCAMS and 72.47% in HMUCH. Disease stage distribution differed between cohorts, with a higher proportion of stage III disease in the CHCAMS cohort (40.4%) compared with the HMUCH cohort (22.9%).

**TABLE 1:** Baseline characteristics of patients with LS‐SCLC in two cohorts.

Characteristic	CHCAMS cohort	HMUCH cohort
**Patients, *n***	245	109
**WSIs, *n***	245	109
**Age, years**		
Mean (SD)	56.57 (10.06)	59.26 (7.81)
Median (IQR)	56 (50–64)	61 (54–65)
**Sex, *n* (%)**		
Male	174 (71.02)	77 (70.64)
Female	71 (28.98)	32 (29.36)
**Smoking, *n* (%)**		
Yes	158 (64.49)	79 (72.47)
No	87 (35.51)	30 (27.53)
**PCI, *n* (%)**		
Yes	57 (23.27)	5 (4.59)
No	188 (76.73)	104 (95.41)
**Brain metastasis during follow‐up, *n* (%)**		
Yes	21 (8.57)	21 (19.27)
No	224 (91.43)	88 (80.73)
**AJCC Stage, *n* (%)**		
I	76 (31.02)	49 (44.95)
II	70 (28.57)	35 (32.11)
III	99 (40.41)	25 (22.94)
**OS, Months**		
Mean (SD)	56.25 (37.38)	45.69 (29.33)
Median (IQR)	47.00 (26.00–80.50)	44.00 (21.00–70.00)
**DFS, Months**		
Mean (SD)	43.31 (39.96)	45.53 (29.11)
Median (IQR)	35.00 (10.00–67.50)	44.00 (21.00–70.00)
**Time to brain metastasis, Months** ^a^		
Mean (SD)	19.32 (24.38)	23.14 (21.10)
Median (IQR)	12.00 (6.00–24.50)	18.00 (12.00–27.00)
**Treatment strategies, *n* (%)**		
Surgery alone	5 (2.04)	33 (30.28)
Postoperative chemoradiotherapy	187 (76.33)	59 (54.13)
Preoperative chemoradiotherapy	24 (9.80)	17 (15.60)
Unknown	29 (11.84)	0 (0.00)

*Note*: Data are presented as number (percentage) for categorical variables and mean (standard deviation) or median (interquartile range) for continuous variables as appropriate.

Abbreviations: LS‐SCLC, Limited‐stage small cell lung cancer; WSIs: Whole‐slide images; PCI: Prophylactic cranial irradiation; AJCC: American Joint Committee on Cancer; IQR: Interquartile range; DFS, Disease‐free survival; OS, Overall survival.

^a^ Calculated only for patients who developed brain metastasis during follow‐up

Because all enrolled patients had surgically resected tumor specimens available for WSI analysis, the cohort was enriched for patients treated with surgery‐based multimodality strategies. Primary treatment strategies are summarized in Table 1. In the CHCAMS cohort, 187 patients received postoperative chemoradiotherapy, 24 received preoperative chemoradiotherapy, 5 underwent surgery alone, and treatment information was unavailable for 29 patients. In the HMUCH cohort, 59 patients received postoperative chemoradiotherapy, 17 received preoperative chemoradiotherapy, and 33 underwent surgery alone.

Among all participants, 292 patients (82.5%) did not receive PCI, while 62 patients (17.5%) underwent PCI treatment. During follow‐up, 42 patients (11.9%) developed radiologically confirmed BM, whereas 312 patients (88.1%) remained free of BM. Among the patients who subsequently developed BM, three had received PCI before radiological detection of intracranial disease. The relatively low PCI rate and crude BM incidence should be interpreted in the context of this retrospective, surgically resected, pathology‐slide‐available cohort, rather than as representative estimates for all LS‐SCLC patients treated with definitive chemoradiotherapy.

For binary model development and direct discrimination assessment, we constructed a high‐confidence endpoint subset among PCI‐naïve patients. Patients who developed radiologically confirmed BM during follow‐up regardless of whether BM occurred as an isolated intracranial relapse or concurrently with extracranial progression were designated as true‐positive cases, whereas patients with DFS ≥5 years and no evidence of metastasis during the follow‐up period were designated as true‐negative cases. This definition was intended to reduce endpoint misclassification in the retrospective setting. Using these criteria, 70 eligible patients were identified from the CHCAMS cohort and 51 from the HMUCH cohort. The baseline clinical characteristics of true‐positive and true‐negative patients are summarized in Table S1. The CHCAMS cohort was randomly divided in a 2:1 ratio into a training set (*n* = 46) for model development and an internal validation set (*n* = 24). The HMUCH cohort (*n* = 51) served as an independent external validation cohort to evaluate the model generalizability. In addition, 62 PCI‐treated patients from both institutions were consolidated into a pooled PCI cohort for exploratory PCI‐related clinical applicability analyses.

### 2.2 Performance of BraMARS for BM Risk Prediction

**FIGURE 2:**
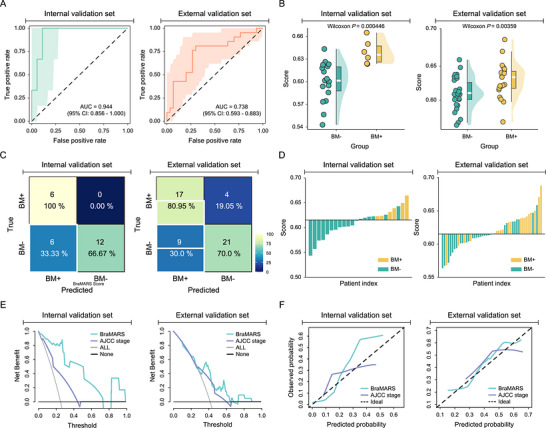
BraMARS performance in brain metastasis risk prediction. (A) Receiver operating characteristic curves showing BraMARS performance in internal and external validation cohorts. (B) Distribution of BraMARS risk scores between patients who developed brain metastasis (BM+) and those who remained metastasis‐free (BM‐). (C) Confusion matrices displaying BraMARS classification performance at optimal threshold in internal and external validation sets. (D) Distribution of patients ranked by BraMARS risk scores, demonstrating the model's ability to stratify patients across the risk spectrum. (E) Decision curve analysis comparing the net benefit of BraMARS‐guided strategy against “treat all” and “treat none” approaches across clinically relevant threshold probabilities. (F) Calibration plots demonstrating agreement between predicted probabilities and observed outcomes, with perfect calibration indicated by the diagonal dashed line.

BraMARS was developed to estimate the future risk of BM from histopathology images. The model was trained on the designated training set and subsequently evaluated through independent validation in both internal and external validation cohorts. As shown in Figure 2, BraMARS demonstrated good discriminatory capability for distinguishing patients who subsequently developed BM from those who remained metastasis‐free during follow‐up. The model achieved an AUC of 0.907 (95% CI: 0.787–1.000) in training, 0.944 (0.856–1.000) in internal validation, and 0.738 (0.593–0.883) in external validation (Figure 2A and Figure S1A). Patients who subsequently developed BM exhibited significantly higher BraMARS risk scores than those who remained free of BM during follow‐up (Figure 2B and Figure S1B). At the optimal risk threshold, determined by maximizing Youden's index in the training set, BraMARS achieved sensitivities of 100% and 81.0%, and specificities of 66.7% and 70.0% in the internal and external validation cohorts, respectively (Figure 2C,D and Figure S1C,D and Table S2). These results suggest that BraMARS may stratify patients according to differing risks of subsequent BM within resected LS‐SCLC patients. Decision curve analysis demonstrated that the BraMARS provided higher net benefit than the “treat all” and “treat none” strategies across a wide range of threshold probabilities from 5% to 70% for the CHCAMS cohort and from 30% to 70% for the HMUCH cohort (Figure 2E). Calibration analysis further revealed good agreement between predicted probabilities and observed BM outcomes. As shown in Figure 2F, the calibration curves for both cohorts generally followed the diagonal reference line, indicating the reliability of BraMARS for individualized BM risk estimation.

### 2.3 Correlation Between BraMARS Risk Stratification and Clinical Outcomes

**FIGURE 3:**
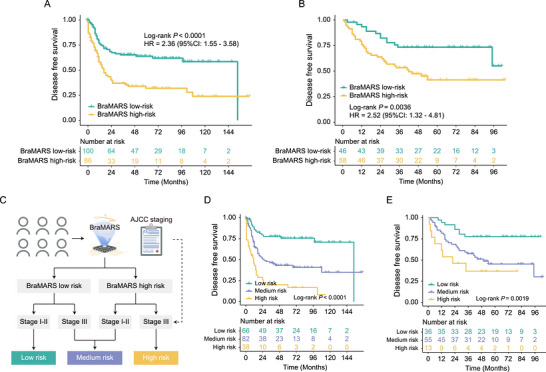
Prognostic value of BraMARS risk stratification. (A‐B) Kaplan‐Meier curves for disease‐free survival in CHCAMS (A) and HMUCH (B) cohorts, stratified by BraMARS risk groups. (C) Integration framework combining BraMARS risk scores with AJCC staging system to create a refined three‐category risk classification. (D,E) Survival analysis of the combined three‐category risk classification in CHCAMS (D) and HMUCH (E) cohorts for disease‐free survival, showing enhanced prognostic stratification.

The prognostic value of BraMARS was evaluated among patients who did not receive PCI across both the CHCAMS and HMUCH cohorts. Patients were categorized into high‐risk and low‐risk groups according to BraMARS predictions. BraMARS classified 88 patients (46.8%) as high‐risk and 100 patients (53.2%) as low‐risk in the CHCAMS cohort, while in the HMUCH cohort, 58 patients (55.8%) were classified as high‐risk and 46 patients (44.2%) as low‐risk. In the CHCAMS cohort, patients classified as high‐risk by BraMARS showed significantly worse DFS compared to low‐risk patients (HR, 2.36; 95% CI, 1.55‐3.58; log‐rank *p* < 0.0001) (Figure 3A). This prognostic performance was also observed in the external HMUCH cohort, where high‐risk patients similarly had shorter DFS (HR = 2.52, 95% CI: 1.32–4.81; log‐rank *p* = 0.0036) (Figure 3B). Multivariable Cox regression analyses adjusting for established clinical factors confirmed that BraMARS high‐risk classification remained independently associated with worse DFS in both the CHCAMS cohort (HR, 2.38; 95% CI, 1.56–3.63; *p* < 0.001) and the HMUCH cohort (HR, 2.47; 95% CI, 1.27–4.81; *p* = 0.0078; Table S3). Beyond DFS, BraMARS also demonstrated robust prognostic value for OS and BMFS. In the CHCAMS cohort, high‐risk patients exhibited significantly shorter OS (HR, 2.19, 95% CI, 1.39–3.46, *p* < 0.001) and BMFS (HR, 9.82, 95% CI, 2.23–43.29, *p* = 0.0026) compared with low‐risk patients. Consistent findings were observed in the HMUCH cohort for both OS (HR, 2.77, 95% CI, 1.43–5.36, *p* = 0.0025) and BMFS (HR, 4.31, 95% CI, 1.45–12.87, *p* = 0.0088) (Figure S2). Multivariable Cox regression analyses further demonstrated that BraMARS risk classification remained independently associated with both OS and BMFS after adjustment for clinical factors in both cohorts (Tables S4 and S5).

We next evaluated whether BraMARS could complement conventional AJCC staging. Combining BraMARS‐predicted risk with conventional AJCC staging established a refined three‐category risk classification system (Figure 3C). In both the CHCAMS and HMUCH cohorts, this integrated classification improved the separation of DFS curves (log‐rank *p* < 0.0001 and *p* = 0.0019, respectively; Figure 3D,E). Patients with both BraMARS high‐risk classification and stage III disease had the poorest outcomes, while those with BraMARS low‐risk classification and stage I‐II disease had the most favorable outcomes. Consistently, the integrated three‐category classification also provided enhanced prognostic stratification for OS and BMFS in both cohorts (Figure S3). Importantly, BraMARS retained prognostic value within AJCC stage subgroups, identifying patients with unfavorable outcomes even among early‐stage patients who might otherwise be considered lower risk based on conventional staging alone (Figures S4 and S5). These findings support the complementary value of histopathology‐based deep learning assessment beyond conventional clinical staging.

### 2.4 Exploratory Clinical Applicability Simulation of BraMARS‐Assisted PCI Allocation

**FIGURE 4:**
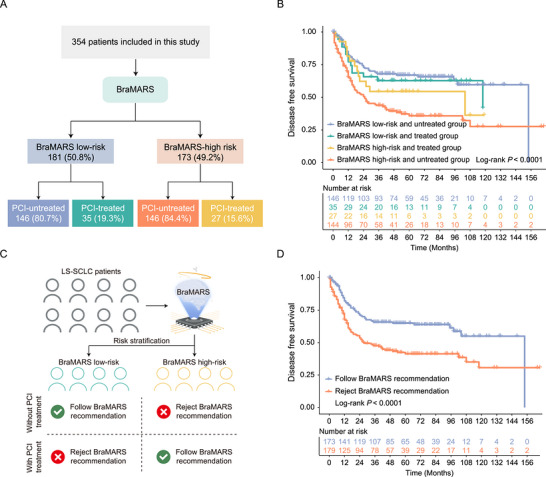
Exploratory retrospective associations between BraMARS risk stratification and PCI exposure. (A) Simulation analysis of BraMARS implementation showing potential reduction in unnecessary PCI treatment and improved coverage for high‐risk patients. (B) Kaplan‐Meier curves for disease‐free survival stratified by BraMARS risk groups and PCI treatment status. (C) Proposed clinical workflow for BraMARS‐assisted PCI decision‐making in LS‐SCLC management. (D) Disease‐free survival comparison between patients managed according to BraMARS recommendations versus those treated contrary to BraMARS guidance.

To explore the potential clinical applicability of BraMARS for individualized PCI risk discussion, we performed an exploratory retrospective simulation using the pooled cohort of 62 PCI‐treated patients and 292 untreated patients. BraMARS risk scores were generated from pretreatment pathological images, and patients were stratified according to BraMARS‐predicted BM risk and actual PCI treatment status. Combined with patients who did not receive PCI therapy (untreated group), all cases were stratified into four distinct categories based on BraMARS predictions and actual treatment received (Figure 4A): 1) patients identified as high‐risk by BraMARS who subsequently received PCI (predicted high‐risk treated group); 2) patients predicted by BraMARS to be at low‐risk for BM but who still received PCI (predicted low‐risk treated group); 3) patients predicted by BraMARS to be at high‐risk for BM but did not receive PCI (predicted high‐risk untreated group); 4) patients predicted by BraMARS to be at low‐risk for BM and did not receive PCI (predicted low‐risk untreated group). As shown in Figure 4A, the simulation revealed that BraMARS‐based stratification could potentially prevent unnecessary PCI in 19.3% of patients (*n* = 35, BraMARS low‐risk treated) without compromising outcomes, while identifying 84.4% of high‐risk patients (*n* = 146, BraMARS high‐risk untreated) who are candidates for PCI but would likely be overlooked under conventional protocols. These findings suggest that BraMARS could provide a risk‐stratified framework for identifying patients with discordance between predicted BM risk and actual PCI use. Kaplan–Meier analysis showed that predicted high‐risk patients who did not receive PCI had worse DFS than predicted high‐risk patients who received PCI (log‐rank *p* < 0.0001, Figure 4B). In contrast, among predicted low‐risk patients, DFS was relatively comparable between PCI‐treated and untreated groups (Figure 4B). Parallel analyses using BMFS as the endpoint yielded concordant results, with predicted high‐risk patients without PCI exhibiting shorter BMFS than predicted high‐risk patients who received PCI, whereas predicted low‐risk patients showed comparable BMFS regardless of PCI status (Figure S6A).

We further compared patients whose actual management was concordant with BraMARS‐based recommendations with those whose management was discordant (Figure 4C). The adherence group, including predicted low‐risk patients who did not receive PCI and predicted high‐risk patients who received PCI, showed significantly better DFS compared to the non‐adherence group, including predicted low‐risk patients who received PCI and predicted high‐risk patients who did not receive PCI (log‐rank *p* < 0.0001, Figure 4D). A similar benefit was observed for BMFS, with superior brain metastasis‐free outcomes in the BraMARS‐concordant group compared with the discordant group (Figure S6B). These exploratory findings indicate that BraMARS may help refine PCI‐related risk discussions by identifying patients with elevated BM risk who may merit consideration of intracranial preventive strategies, while potentially sparing predicted low‐risk patients from unnecessary treatment‐related toxicity.

### 2.5 Pathological Interpretability of BraMARS Predictions

**FIGURE 5:**
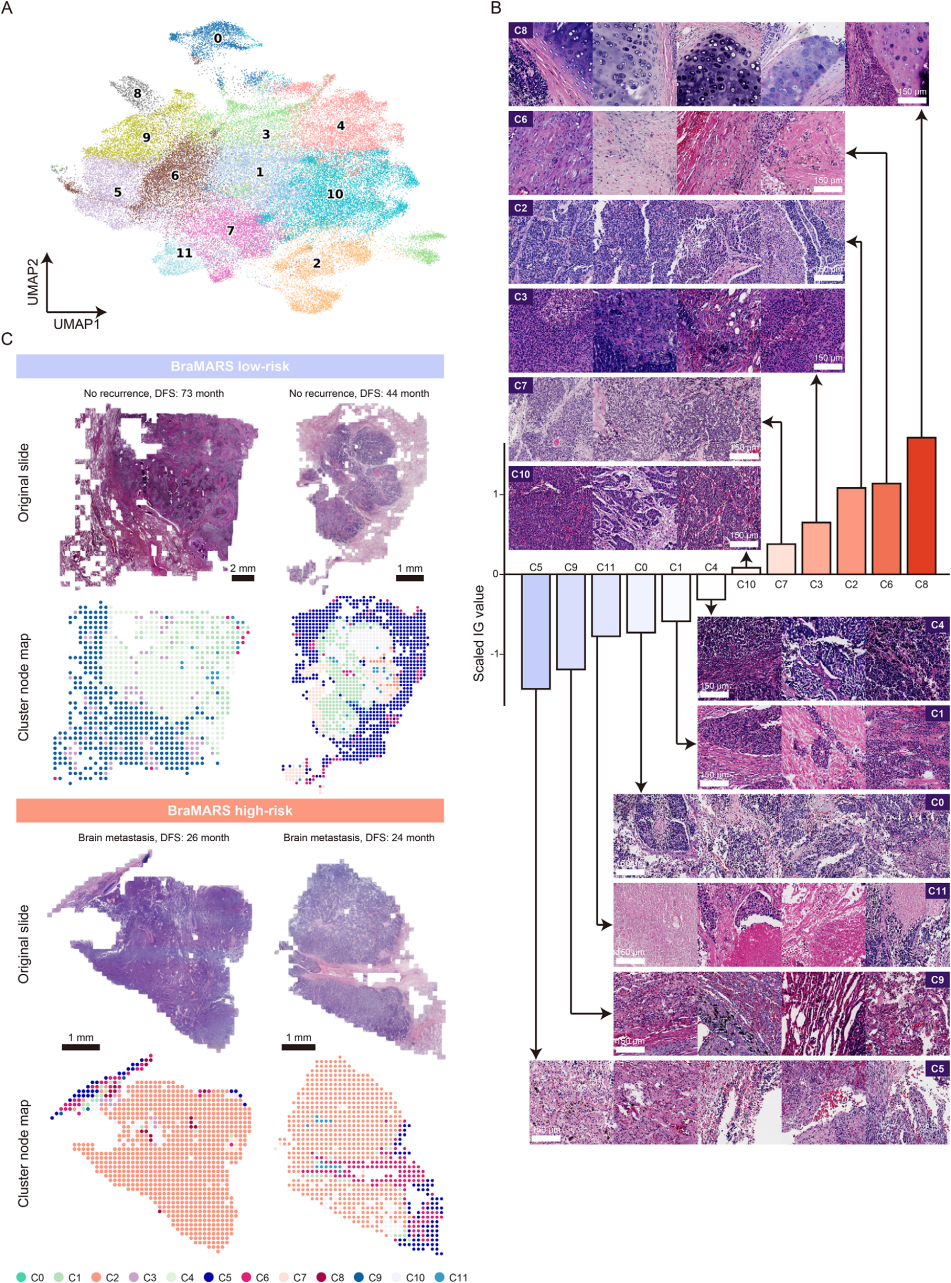
Histopathological interpretability of BraMARS predictions. (A) UMAP visualization of 12 histopathological clusters derived from deep learning features. (B) Integrated gradients heatmap showing contribution of different histopathological clusters to BraMARS risk predictions. (C) Representative whole‐slide images with cluster annotations and corresponding clinical outcomes, demonstrating spatial distribution patterns associated with high‐risk and low‐risk predictions.

To quantitatively analyze the relationship between histopathological features and BraMARS risk, we first clustered the WSI patches into 12 distinct histopathological clusters (HCs) based on their learned morphological feature representations (Figure 5A and Figure S7). We then calculated the Integrated Gradients (IG) values for each patch and mapped these values onto the corresponding HCs to assess their contribution to the model's risk prediction (Figure 5B). Pathological evaluation revealed that HCs associated with high‐risk BraMARS predictions (HC8, HC6, and HC2) were characterized by features commonly seen in aggressive tumors. Specifically, HC2 regions exhibited high tumor cellularity with nuclear crowding, pleomorphism, and frequent mitotic figures. HC6 and HC8 displayed prominent stromal activation, featuring proliferating fibroblasts within a dense extracellular matrix and fragments of pulmonary cartilage. These histopathological patterns are well‐established markers of invasive potential and metastatic competence and support the classification of these areas as high‐risk by BraMARS, correlating with an elevated risk of subsequent BM. Conversely, HCs associated with BraMARS low‐risk predictions (HC5, HC9, HC1 and HC11) presented features associated with favorable prognosis. HC5 and HC9 maintained relatively preserved lung architecture with identifiable alveolar septa and bronchial epithelium. HC1 showed significant immune cell infiltration, predominantly lymphocytes, suggesting active anti‐tumor immunity. HC11 contained extensive necrotic areas, which in this context may indicate response to therapy or impaired tumor viability. Furthermore, the spatial distribution and prevalence of these HCs differed markedly across patients with different BraMARS risk classifications. High‐risk patients demonstrated a clear clustering of aggressive histology (HC2, HC6, HC8) with correspondingly high IG values, indicating these regions are primary drivers of the poor prognosis prediction (Figure 5C). In contrast, low‐risk patients exhibited a more heterogeneous tissue landscape dominated by favorable HCs, with a dispersed spatial pattern that correlates with better clinical outcomes, as evidenced by the significantly longer disease‐free survival in representative cases (Figure 5C). This comprehensive pathological analysis confirms that BraMARS risk stratification captures fundamental differences in tumor microenvironment organization, cellular composition, and architectural patterns.

### 2.6 Biological Basis of BraMARS Risk Stratification

**FIGURE 6:**
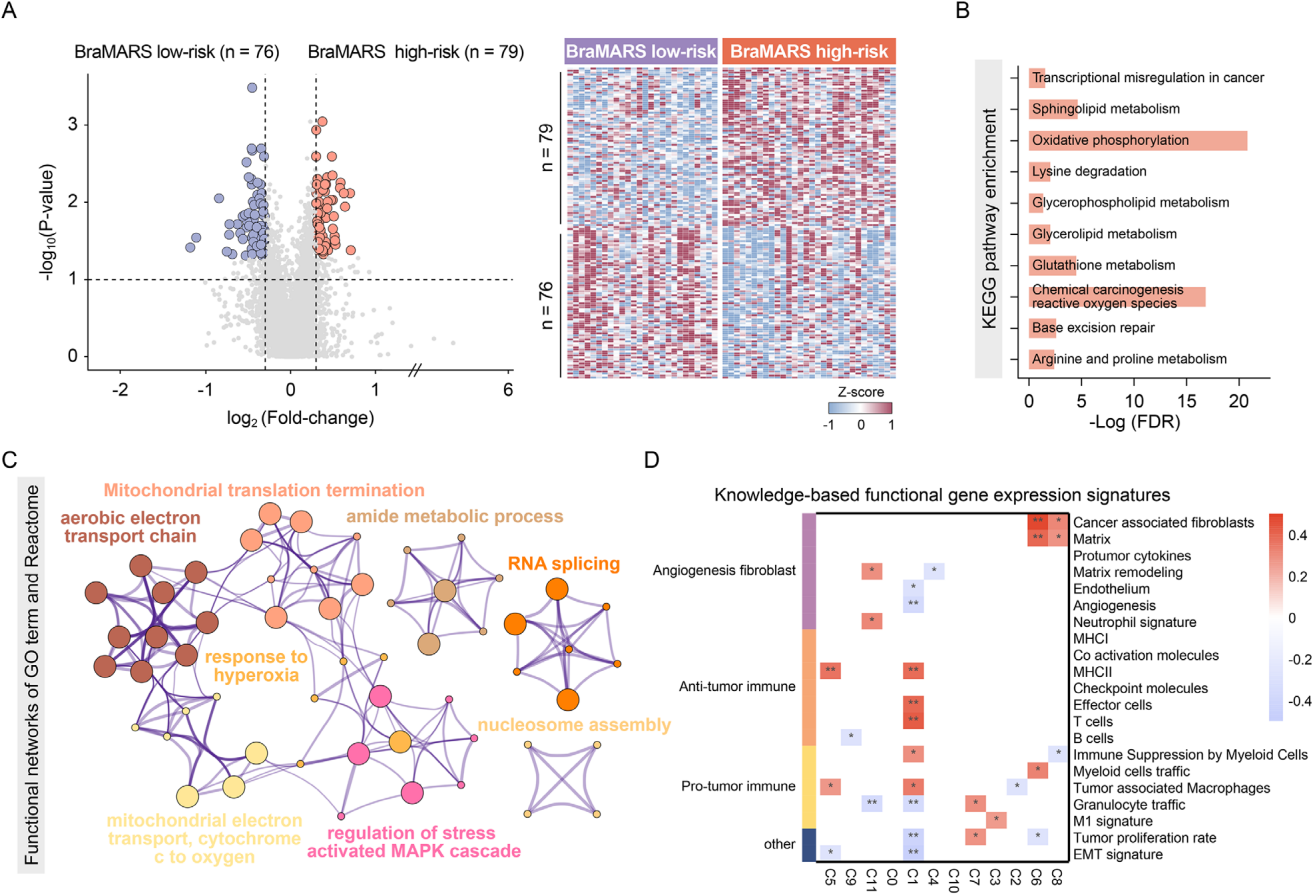
Biological basis of BraMARS risk stratification. (A) Volcano plot and unsupervised clustering heatmap displaying differentially expressed proteins between BraMARS high‐risk and low‐risk groups. (B) Functional enrichment analysis of KEGG pathways. (C) Functional networks of Gene Ontology Biological Processes. (D) Correlation heatmap between histopathological clusters and functional gene expression signatures, revealing associations with tumor microenvironment components.

To explore the underlying biological basis of BraMARS predictions, we performed proteomic analysis on tumor samples from 56 LS‐SCLC patients. Based on BraMARS' predicted risk scores, these patients were categorized into high‐risk and low‐risk groups. Differential protein expression analysis identified 79 proteins significantly upregulated in the BraMARS high‐risk group and 76 proteins upregulated in the low‐risk group (Figure 6A). Unsupervised clustering analysis demonstrated that the expression patterns of these differentially expressed proteins (DEPs) effectively segregated the two risk groups (Figure 6A), suggesting that BraMARS‐defined risk stratification was associated with distinct molecular profiles. Functional enrichment analysis of differentially expressed proteins further revealed biological pathways associated with BraMARS risk classification (Figure 6B). These DEPs were significantly enriched in mitochondrial energy production pathways, transcriptional regulation in cancer, reactive oxygen species (ROS) detoxification, DNA repair (Figure 6C). These findings suggest that BraMARS high‐risk tumors may be characterized by a metabolically active and stress‐adapted phenotype.

To bridge molecular features with histological patterns, we performed ssGSEA using 29 functionally defined gene expression signatures and examined their associations with histopathological clusters. This analysis revealed coherent relationships between BraMARS‐associated histological patterns and tumor microenvironment states (Figure 6D). High‐risk histological clusters (HC6 and HC8) were positively associated with cancer‐associated fibroblasts, matrix remodeling, and pro‐tumor immune signatures, while low‐risk clusters (HC1 and HC5) were associated with immune‐related features, including T cell and B cell signatures. Additionally, high‐risk group exhibited higher scores for tumor proliferation and epithelial‐mesenchymal transition (EMT) signature, consistent with a more invasive and microenvironmentally activated tumor state [13].

## 3 Discussion

In this study, we developed BraMARS, an interpretable deep learning model that estimates the future risk of BM from routinely available H&E‐stained WSIs in patients with resected LS‐SCLC. BraMARS demonstrated robust discriminatory performance across independent cohorts and stratified patients into groups with significantly different DFS, OS, and BMFS. Importantly, BraMARS provided prognostic information beyond conventional AJCC staging, supporting the complementary value of histopathology‐based risk assessment for identifying patients with elevated intracranial metastatic risk.

Although PCI reduces BM incidence and may improve survival in selected LS‐SCLC [14, 15], its clinical applicability remains challenging because of potential long‐term neurocognitive toxicity and the lack of individualized BM risk assessment. A recent single‐institution cohort study suggested that the benefits of PCI for unselected patients with LS‐SCLC are limited due to the neurocognitive toxic effects associated with whole‐brain radiation therapy [6]. Therefore, current PCI guidelines, while demonstrating population‐level benefit, inevitably lead to both overtreatment of low‐risk patients and undertreatment of high‐risk individuals [16]. Previous studies have focused on the role of conventional clinical factors, such as clinical staging, nodal status and age, in predicting brain metastasis [17, 18, 19]. While these factors provide some prognostic information, they lack the precision required for personalized decision‐making [20]. Emerging imaging‐based approaches show promise but are often constrained to post‐metastasis settings and do not address the need for preemptive risk assessment [12, 21, 22]. BraMARS addresses this critical gap by extracting risk‐relevant information from routinely available histopathological images, thereby providing a candidate framework for individualized BM risk stratification. The model demonstrated robust and generalizable performance across independent cohorts. In exploratory PCI‐related analyses, BraMARS identified predicted low‐risk patients who had received PCI and predicted high‐risk patients who had not received PCI. These findings suggest that BraMARS may help refine PCI‐related risk discussions by identifying patients with elevated BM risk who may warrant closer intracranial surveillance or consideration of preventive strategies, while potentially sparing predicted low‐risk patients from unnecessary treatment‐related toxicity. However, PCI administration was not randomized in this retrospective cohort, and treatment decisions were likely influenced by clinical factors, patient preference, physician judgment, postoperative treatment pathways, and evolving MRI surveillance practices. Therefore, the PCI‐related simulation should be interpreted as hypothesis‐generating rather than as causal evidence that BraMARS‐guided PCI allocation improves clinical outcomes.

The pathological interpretability analysis showed that BraMARS high‐risk predictions were associated with histopathological patterns indicative of aggressive tumor behavior, including high tumor cellularity, nuclear pleomorphism, and stromal activation. These morphological patterns were complemented by proteomic signatures highlighting enhanced mitochondrial metabolism, ROS detoxification, and upregulated DNA repair pathways in high‐risk tumors. The enrichment of mitochondrial energy production and oxidative stress defense pathways aligns with the known metabolic dependencies of aggressive SCLC variants [23, 24, 25], while upregulated DNA repair programs may contribute to treatment resistance and metastatic competence [26, 27, 28, 29]. Previous studies have implicated SCLC‐derived PLGF in blood‐brain barrier disruption and transendothelial migration, whereas more recent studies suggest that SCLC cells may recruit reactive astrocytes through Reelin‐dependent signaling and co‐opt neuron‐astrocyte developmental programs to support metastatic outgrowth in the brain [30, 31]. In this context, the enrichment of mitochondrial metabolism, oxidative stress defense, and DNA repair programs in the BraMARS high‐risk group is consistent with a tumor state characterized by metabolic flexibility and stress tolerance, which may favor dissemination and colonization [3, 13]. Collectively, these findings indicate that BraMARS captures fundamental biological divergence within SCLC, distinguishing tumors with a pro‐metastatic, metabolically active, and stress‐adapted phenotype from those with a more indolent molecular and microenvironmental profile [32, 33]. Similarly, the association of high‐risk histological clusters with stromal activation, fibroblast‐rich niches, matrix remodeling, and EMT‐related programs supports a more invasive and microenvironmentally adaptive phenotype [13, 34]. Collectively, these findings support the biological plausibility of BraMARS and suggest that the high‐risk and low‐risk groups may represent distinct tumor microenvironmental states within SCLC.

Several limitations should be acknowledged. First, BraMARS was developed and validated in a surgically resected, pathology‐slide‐available LS‐SCLC cohort. The model has not been validated in diagnostic biopsy specimens or in unresected stage III LS‐SCLC treated with definitive chemoradiotherapy, which represents the predominant LS‐SCLC population. Although the model input consists of H&E‐stained histopathological images and is therefore technically compatible with biopsy specimens in principle, biopsy‐based deployment has not yet been validated. Biopsy specimens differ from resection specimens in tissue area, tumor‐cell fraction, stromal representation, crush artifact, necrosis, and sampling of intratumoral heterogeneity. These differences may introduce specimen‐related domain shift and could compromise model calibration or discrimination. Therefore, BraMARS should not currently be applied to biopsy specimens or unresected chemoradiotherapy‐treated LS‐SCLC patients without dedicated validation. Future studies should therefore evaluate paired biopsy and resection specimens, define biopsy‐specific quality‐control and minimum‐tissue requirements, and externally validate a locked model in independent definitive‐chemoradiotherapy cohorts before any biopsy‐based clinical implementation is considered. Second, PCI administration was not randomized, and the PCI‐related simulation may be affected by treatment‐selection bias and unmeasured confounding. Therefore, this analysis should be regarded as exploratory and hypothesis‐generating rather than causal evidence that BraMARS‐guided PCI allocation improves clinical outcomes. Third, proteomic profiling was available only in a subset of patients, limiting statistical power for pathway‐level inference and mechanistic interpretation. These molecular findings should therefore be regarded as supportive biological context rather than definitive mechanistic evidence. Future prospective multicenter studies incorporating standardized MRI surveillance, detailed PCI decision records, biopsy and resection specimens, multi‐omic profiling, and functional validation are needed to determine whether BraMARS can improve individualized BM prevention strategies in LS‐SCLC.

In conclusion, BraMARS provides an interpretable histopathology‐based framework for estimating future BM risk in resected, pathology‐slide‐available LS‐SCLC. BraMARS complements conventional staging, stratifies clinically relevant survival outcomes, and is supported by biologically plausible histopathological and proteomic correlates. Exploratory PCI‐related analyses suggest that BraMARS may inform individualized PCI risk discussion, particularly when BMFS is used as the most directly relevant endpoint. Prospective validation in biopsy specimens, unresected definitive chemoradiotherapy‐treated LS‐SCLC cohorts, and prospectively followed populations is required before BraMARS can be used to influence PCI or MRI‐surveillance decisions.

## 4 Materials and Methods

### 4.1 Ethics Statement

This study was conducted in accordance with the Declaration of Helsinki guidelines and ethics approval was obtained from the Cancer Hospital, Chinese Academy of Medical Sciences (approval number 22/250‐3452) and Harbin Medical University Cancer Hospital (approval number YD2025‐14). The requirement for informed consent was waived by both ethics committees because of the retrospective nature of the study.

### 4.2 Study Participants and Patient Cohorts

In this retrospective study, a total of 354 patients with limited‐stage small cell lung cancer (LS‐SCLC) were enrolled from two independent medical institutions, including 245 patients from the Cancer Hospital, Chinese Academy of Medical Sciences (CHCAMS cohort) between January 2005 and December 2016, and 109 patients from Harbin Medical University Cancer Hospital (HMUCH cohort) between December 2014 and December 2020 (Figure 1A). Inclusion criteria were as follows: (1) histologically confirmed LS‐SCLC at initial presentation; (2) R0 surgical resection with available H&E‐stained whole‐slide images (WSIs) generated from surgically resected formalin‐fixed paraffin‐embedded (FFPE) tumor specimens; and (3) clinicopathological and follow‐up data. Exclusion criteria were: (1) radiologically evident brain metastasis at initial diagnosis; (2) prior history of cranial irradiation or neurosurgery before SCLC diagnosis; and (3) unavailable H&E‐stained pathological slides, incomplete clinical data, or inadequate WSI quality.

The primary endpoint was radiologically confirmed BM during follow‐up. Patients classified as BM‐positive in this study were those who developed brain metastasis after study entry, rather than patients with pre‐existing intracranial metastasis at baseline. Brain metastasis‐free survival (BMFS) was defined as the time from diagnosis to radiologically confirmed brain metastasis or last follow‐up, with death treated as a competing event where appropriate. Secondary endpoints included overall survival (OS), defined as the time from diagnosis to death from any cause or last follow‐up and disease‐free survival (DFS), defined as the time from diagnosis to disease recurrence or death, or last follow‐up.

### 4.3 Digital WSI Acquisition and Preprocessing

FFPE tumor sections from surgically resected specimens from all study participants were stained with H&E and digitized into WSIs at 20× resolution using digital slide scanners. The acquired WSIs were segmented into non‐overlapping tiles of 224 × 224 pixels at 2 µm/pixel. Otsu's thresholding method was applied to filter out artifacts and non‐tissue regions, retaining only tiles containing ≥50% tissue area for subsequent analysis.

### 4.4 WSI‐Based Deep Learning Model Development

We developed a deep learning model based on Structured State Space Models for Multiple Instance Learning (S4MIL) [35] for Brain Metastasis Assessment and Risk Stratification, termed BraMARS, to estimate future BM risk from WSIs in PCI‐naïve patients with LS‐SCLC. The deep learning framework, illustrated in Figure 1B, comprises three sequential components: WSI preprocessing and patch‐level feature extraction, S4‐based sequential feature encoding, and multiple instance learning aggregation for slide‐level classification.

### 4.5 Data Preprocessing and Patch‐Level Feature Extraction

To generate robust and informative tile‐level features, each 224 × 224 pixel patch was processed for feature extraction using the CTransPath model which was pretrained on large‐scale publicly available histopathology datasets and integrates the local feature extraction capabilities of Convolutional Neural Networks (CNNs) with the global contextual modeling strengths of Transformers [36]. Finally, this pre‐trained model generated 768‐dimensional morphological feature representations for each patch.

### 4.6 Feature Encoding With S4

The extracted patch‐level features were then processed by the Diagonal Structured State Space Model (S4D) [37]. Neural state space models (SSMs) use continuous‐time state equations to describe the dynamics of input data and model the temporal dependencies:

(1) $$\dot{x}\;\left(t\right)=Ax\left(t\right)+Bu\left(t\right)$$

(2) $$y\;\left(t\right)=Cx\left(t\right)+Du\left(t\right)$$

where *u*(*t*) denotes the input signal, *x*(*t*) is the hidden state, *y*(*t*) is the output signal, and ***A***, ***B***, ***C***, ***D*** are learnable system matrices. For discrete‐time implementation, we adapted the state space equations into a recursive form analogous to recurrent neural networks (RNNs):

(3) $$\;x_{t}=\bar{A}x_{t-1}+\bar{B}u_{t}$$

(4) $$\;y_{t}=\bar{C}x_{t}+\bar{D}u_{t}$$

The S4D variant of the SSM framework simplifies computation by approximating the state matrix ***A*** as diagonal and leveraging Fast Fourier Transforms (FFT) to convert recursive state updates into parallelizable convolutions. The S4D Kernel generates state space kernels by learning parameters for the diagonal matrix ***A*** and state matrices ***B*** and ***C***. The hidden states *x*(*t*) serve as memory units, encoding historical information from the sequence of patch features. HiPPO [38] (high‐order polynomial projection operator) initialization was applied to optimize the model's ability to capture long‐range dependencies within the WSI, which is critical for modeling the spatial propagation of pathological features.

### 4.7 Feature Aggregation and Classification via MIL

The final output signal *y*(*t*) for each patch is generated from skip connections that combine the original input u and the transformed hidden state *x*(*t*). The state matrix ***C*** enhances the nonlinear mapping from states to outputs, prioritizing the most relevant features for prediction, while the matrix ***D*** preserves high‐frequency information to prevent gradient vanishing. The resulting sequence of patch‐level representations *y*(*t*) is then aggregated into a single slide‐level representation using a max‐pooling operation, a standard MIL approach that identifies the most informative patches for final prediction. The aggregated feature vector was then processed through a classifier to generate brain metastasis risk predictions.

### 4.8 Tissue Proteomics Analysis

Proteomic profiling was performed on tumor specimens from 56 SCLC patients and were obtained from China National Center for Bioinformation/Beijing Institute of Genomics, Chinese Academy of Sciences (https://ngdc.cncb.ac.cn/omix: accession no. OMIX007230) [39]. Protein abundance values were normalized using median centering followed by log2 transformation. Proteins with missing values in more than 25% of samples were excluded from subsequent analysis. Remaining missing values were imputed using the k‐nearest neighbor (k‐NN) algorithm in the DreamAI R package (https://github.com/WangLab‐MSSM/DreamAI) [40]. Differentially expressed proteins (DEPs) between the BraMARS low‐risk and high‐risk groups were identified via the Wilcoxon test, with significant DEPs defined by *p*‐values < 0.1 and log2 (fold change) > 0.3 or < (−0.3).

### 4.9 Functional Characterization of Differentially Expressed Proteins

Functional enrichment analysis of differentially expressed proteins was conducted across Kyoto Encyclopedia of Genes and Genomes (KEGG) pathways and Gene Ontology Biological Processes (GO BP) using the clusterProfiler R package [41]. The enrichment functional networks of GO BP terms were constructed using Metascape (http://metascape.org/) [42]. 29 knowledge‐based functional gene expression signatures (FGES) representing the major functional components and immune, stromal, and other cellular populations of the tumor were obtained from Bagaev's study [43]. Single‐sample gene set enrichment analysis (ssGSEA) of 29 FGES were performed using GSVA (version 2.0.1).

### 4.10 Pathological Visualization and Interpretation Analysis

To elucidate the histopathological basis of BraMARS predictions and enhance model interpretability, we employed an integrated approach combining computational pathology with feature attribution mapping. The Integrated Gradients (IG) method was applied to quantify the contribution of individual image patches to the final risk prediction. The IG values were calculated as follows:

(5)

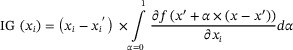

where, *f*(*x*) represents the BraMARS, *x* and *x*′ denote the feature vectors of input patches and baseline reference respectively, and α represents the interpolation step. Here, we set zero vector for baseline feature reference *x*′. Patches with higher IG values were identified as stronger contributors to high‐risk predictions, while those with lower values contributed more to low‐risk classifications.

To systematically analyze the relationship between morphological patterns and model predictions, we performed unsupervised clustering using the K‐means clustering algorithm of all image patches based on their deep learning‐derived feature representations, and the cluster number was determined by combining the Calinski–Harabasz index and Davies–Bouldin index.

To explore associations between histopathological features and their underlying molecular phenotypes, we represented each patient as a feature matrix comprising the proportional distribution of histopathological clusters:

(6) $$P=\left[p_{1},p_{2},\text{…},p_{n}\right]$$

(7) $$p_{i}=\left\{\frac{c_{i,0}}{m_{i}},\frac{c_{i,1}}{m_{i}},\text{…},\frac{c_{i,11}}{m_{i}}\right\}\;$$

where *n* represents the number of patients, while *c*_*i*,*j*_ and *m_i_* represent the number of patches from patient *i* assigned to cluster *j* and the total number of patches from patient *i*, respectively. We subsequently quantified associations between ssGSEA scores of 29 FGES and histopathological clusters by computing Pearson correlation coefficients with the matrix ***P***.

### 4.11 Implementation Details

The BraMARS deep learning model was implemented and trained on data from the CHCAMS cohort using the following computational framework. Model optimization was performed using the AdamW optimizer with a batch size of 1. The training protocol used an initial learning rate of 1 × 10^−^^5^, weight decay of 1 × 10^−^^4^, and extended over 200 epochs to ensure convergence. A cosine annealing schedule with restarts was implemented to dynamically adjust learning rates throughout training. The binary cross‐entropy loss with logits (BCEWithLogitsLoss) was used as the objective function. Our architectural implementation builds upon the open‐source S4MIL framework. All computational experiments were conducted on a Windows workstation equipped with an NVIDIA GeForce RTX 4060 GPU, using PyTorch (version 2.1.0) as the deep learning framework.

### 4.12 Statistical Analysis

All statistical analyses were performed using R software (version 4.1.3) and relevant R packages. Continuous variables were summarized as means with standard deviations or medians with interquartile ranges (IQR), and categorical variables were presented as frequencies and percentages. Group comparisons for continuous variables were conducted using the Wilcoxon rank‐sum test, while categorical variables were compared using Fisher's exact test or the Chi‐squared test, as appropriate. Survival curves were generated using the Kaplan–Meier method, and differences between survival distributions were assessed using the log‐rank test with the R package ‘survminer’ (version 0.4.9). The prognostic value of BraMARS was evaluated using univariate and multivariate Cox proportional hazards regression models, with results reported as hazard ratios (HR) and corresponding 95% confidence intervals (CI). The predictive performance of BraMARS was quantified by the area under the receiver operating characteristic curve (AUC). The optimal probability threshold was determined by maximizing Youden's J statistic in the training set. Model performance was further evaluated using accuracy, F1‐score, recall (sensitivity), and specificity. A two‐sided p‐value < 0.05 was considered statistically significant.

## Author Contributions

M.Z. and L.Y. supervised and designed the study. S.L.L., F.Y., and L.Y. contributed materials and clinical expertise. T.L.W. and Z.J.Y. trained and developed the artificial intelligence model. Z.J.Y., T.L.W., Z.C.Z., Y.B.Z., B.Y., S.S.G., and Y.C. performed analysis and interpretation of data. T.L.W., Z.J.Y., and Z.C.Z. performed the initial manuscript preparation. M.Z. and L.Y. critically revised the manuscript. All authors accept the final responsibility to submit for publication and take responsibility for the contents of the manuscript.

## Patient Consent Statement

Informed consent was waived for this retrospective study by both ethics committees.

## Conflicts of Interest

The authors have declared no conflicts of interest.

## Code Availability Statement

The code of this work is available at https://github.com/ZhoulabCPH/BraMARS.

## Supporting information




**Supporting File**: advs77075‐sup‐0001‐SuppMat.docx.

## Data Availability

The whole‐slide histopathology images and associated clinical data generated and analyzed during this study are not publicly available due to patient privacy concerns and institutional data protection policies. These data may be made available to qualified researchers for non‐commercial research purposes upon reasonable request to the corresponding author, Dr. Lin Yang (yanglin@cicams.ac.cn) and Dr. Shilong Liu (liushilong1984@hrbmu.edu.cn), subject to institutional data transfer agreement and ethical approval. The mass spectrometry proteomics data have been deposited in the OMIX database (https://ngdc.cncb.ac.cn/omix/) under accession code OMIX007230.
