# Supplementary material for: BraMARS: An Interpretable Histopathology‐Driven Deep Learning Model for Brain Metastasis Risk Stratification in Surgically Resected Limited‐Stage SCLC
Source: Adv Sci (Weinh). 2026 Aug 14:e77075. Online ahead of print. doi: 10.1002/advs.77075 (PMC13474182; doi:10.1002/advs.77075)
Supplement: Supplementary file 1 [file ADVS-9999-e77075-s001.docx]

**Supporting Information**

**BraMARS: an interpretable histopathology-driven deep learning model for brain metastasis risk stratification in surgically resected limited-stage SCLC**

*Zijiang Yang^1#^, Taolue Wang^1#^, Shilong Liu^2^**^#^, Zicheng Zhang^1^, Yibo Zhang^1^, Fan Yang^3^, Bo Yu^4^, Shuaishuai Gao^1^, Yu Chen^1^, Lin Yang**^3^*, Meng Zhou^1^**

**Figure S1. BraMARS performance on the training set.** **(A)** Receiver operating characteristic (ROC) curve of BraMARS for brain metastasis (BM) risk prediction. **(B)** Distribution of BraMARS risk scores in patients with (BM+) and without (BM-) subsequent brain metastasis. (**C**) Confusion matrix showing classification performance at the optimal probability threshold. **(D)** Patients ranked by BraMARS risk score.

**Figure S2. Prognostic value of BraMARS risk groups for overall survival and brain metastasis-free survival.** (A-B) Kaplan-Meier curves for overall survival in CHCAMS (A) and HMUCH (B) cohorts, stratified by BraMARS risk groups. (C-D) Kaplan-Meier curves for brain metastasis-free survival in CHCAMS (C) and HMUCH (D) cohorts, stratified by BraMARS risk groups.

**Figure S3. Prognostic performance of the combined three-category risk classification for overall survival and brain metastasis-free survival.** (A-B) Survival analysis of the combined three-category risk classification in CHCAMS (A) and HMUCH (B) cohorts for overall survival. (C)-(D) Survival analysis of the combined three-category risk classification in CHCAMS (C) and HMUCH (D) cohorts for brain metastasis-free survival.

**Figure S4. Performance of BraMARS across AJCC stage subgroups.** ROC curves depicting model performance stratified by AJCC stage (I, II, III).

**Figure S5. Kaplan–Meier survival analysis of BraMARS risk groups within AJCC stage subgroups.** Kaplan–Meier curves comparing survival outcomes between the BraMARS low-risk and high-risk groups within each AJCC stage subgroup. (A–C) Patients with AJCC stage I: disease-free survival (A), overall survival (B), and brain metastasis-free survival (C). (D–F) Patients with AJCC stage II: disease-free survival (D), overall survival (E), and brain metastasis-free survival (F). (G–I) Patients with AJCC stage III: disease-free survival (G), overall survival (H), and brain metastasis-free survival (I).

**Figure S6. Brain metastasis-free survival analysis of BraMARS-guided PCI decision-making.** (A) Kaplan-Meier curves for brain metastasis-free survival stratified by BraMARS risk groups and PCI treatment status. (B) Brain metastasis-free survival comparison between patients managed according to BraMARS recommendations versus those treated contrary to BraMARS guidance.

**Figure S7. Determination of the optimal number of histopathological clusters.** Normalized clustering evaluation metrics (Calinski-Harabasz index, Davies-Bouldin index, and combined score) across different cluster numbers, supporting the selection of 12 histopathological clusters for subsequent analysis.

**Table S1. Clinical characteristics of true-positive and true-negative LS-SCLC patients**

| **Variables** | **CHCAMS cohort (N=70)** | | | **HMUCH cohort (N=51)** | | | |
| --- | --- | --- | --- | --- | --- | --- | --- |
|  | True-positive (N=18) | True-negative (N=52) | *P value* | | True-positive (N=21) | True-negative (N=30) | *P value* |
| **Age (years)** |  |  |  | |  |  |  |
| Mean (SD) | 61.06 (9.06) | 57.67 (8.88) | 0.303 | | 61.76 (6.08) | 56.87 (8.34) | 0.036 |
| **Gender, *n* (%)** |  |  |  | |  |  |  |
| Male | 16 (88.89) | 31 (59.62) | 0.039 | | 15 (71.43) | 19 (63.33) | 0.763 |
| Female | 2 (11.11) | 21 (40.38) |  | | 6 (28.57) | 11 (36.67) |  |
| **Smoking, *n* (%)** |  |  |  | |  |  |  |
| Yes | 14 (77.78) | 30 (57.69) | 0.163 | | 16 (76.19) | 22 (73.33) | >0.999 |
| No | 4 (22.22) | 22 (42.31) |  | | 5 (23.81) | 8 (26.67) |  |
| **AJCC stage, *n* (%)** |  |  |  | |  |  |  |
| I | 1 (5.55) | 20 (38.46) | 0.002 | | 6 (28.57) | 18 (60.00) | 0.081 |
| II | 5 (27.78) | 20 (38.46) |  | | 9 (42.86) | 8 (26.67) |  |
| III | 12 (66.67) | 12 (23.08) |  | | 6 (28.57) | 4 (13.33) |  |

Note: Data are presented as number (percentage) for categorical variables and mean (standard deviation) for continuous variables as appropriate. True-positive denotes patients who developed brain metastasis, and true-negative denotes patients with disease-free survival ≥5 years without evidence of metastasis. P-values were derived from Wilcoxon rank-sum test for continuous variables and Fisher's exact test for categorical variables. LS-SCLC, limited-stage small cell lung cancer;

**Table S2. Performance of BraMARS for BM risk prediction across different datasets**

| **Dataset** | **F1-Score** | **Specificity** | **Sensitivity** | **Accuracy** |
| --- | --- | --- | --- | --- |
| **Training set** | 0.714 | 0.823 | 0.833 | 0.826 |
| **Internal validation set** | 0.667 | 0.667 | 1.000 | 0.750 |
| **External validation set** | 0.723 | 0.700 | 0.810 | 0.745 |

**Table S3. Univariable and multivariable Cox regression analyses of BraMARS and clinical factors for disease-free survival**

| **Variables** | Univariable analysis |  |  | Multivariable analysis |  |
| --- | --- | --- | --- | --- | --- |
|  | Hazard ratio (95% CI) | *P* value |  | Hazard ratio (95% CI) | *P* value |
| **CHCAMS cohort** |  |  |  |  |  |
| BraMARS (high risk vs. low risk) | 2.36 (1.55-3.58) | **<0.001** |  | 2.38 (1.56-3.63) | **<0.001** |
| Age | 0.98 (0.96-1.00) | 0.091 |  | 0.98 (0.96-1.01) | 0.13 |
| Gender (female vs. male) | 0.49 (0.30-0.81) | 0.0047 |  | 0.61 (0.33-1.13) | 0.12 |
| Smoking (yes vs. no) | 1.56 (1.00-2.42) | 0.051 |  | 1.30 (0.74-2.28) | 0.36 |
| AJCC stage (III vs. I&II) | 2.98 (1.97-4.51) | **<0.001** |  | 2.61 (1.71-3.97) | **<0.001** |
| **HMUCH cohort** |  |  |  |  |  |
| BraMARS (high risk vs. low risk) | 2.52 (1.32-4.81) | **0.0051** |  | 2.47 (1.27-4.81) | **0.0078** |
| Age | 1.05 (1.01-1.09) | **0.022** |  | 1.06 (1.02-1.11) | **0.0064** |
| Gender (female vs. male) | 0.71 (0.37-1.38) | 0.31 |  | 0.79 (0.39-1.60) | 0.52 |
| Smoking (yes vs. no) | 1.52 (0.75-3.08) | 0.24 |  | 1.00 (0.47-2.11) | 1 |
| AJCC stage (III vs. I&II) | 1.90 (0.99-3.62) | 0.052 |  | 2.42 (1.22-4.79) | **0.012** |
| Bold values indicate significant P values (<0.05). CI = confidence interval. | | | | | |

**Table S4. Univariable and multivariable Cox regression analyses of BraMARS and clinical factors for overall survival**

| **Variables** | Univariable analysis |  |  | Multivariable analysis |  |
| --- | --- | --- | --- | --- | --- |
|  | Hazard ratio (95% CI) | *P* value |  | Hazard ratio (95% CI) | *P* value |
| **CHCAMS cohort** |  |  |  |  |  |
| BraMARS (high risk vs. low risk) | 2.19 (1.39-3.46) | **<0.001** |  | 2.08 (1.31-3.29) | **0.0018** |
| Age | 1.00 (0.97-1.02) | 0.7 |  | 1.01 (0.98-1.03) | 0.62 |
| Gender (female vs. male) | 0.54 (0.31-0.92) | 0.025 |  | 0.58 (0.29-1.14) | 0.11 |
| Smoking (yes vs. no) | 1.36 (0.84-2.20) | 0.22 |  | 1.06 (0.58-1.94) | 0.86 |
| AJCC stage (III vs. I&II) | 3.09 (1.95-4.90) | **<0.001** |  | 2.93 (1.83-4.69) | **<0.001** |
| **HMUCH cohort** |  |  |  |  |  |
| BraMARS (high risk vs. low risk) | 2.77 (1.43-5.36) | **0.0025** |  | 2.50 (1.27-4.91) | **0.0079** |
| Age | 1.04 (1.00-1.08) | 0.053 |  | 1.06 (1.01-1.10) | 0.012 |
| Gender (female vs. male) | 0.55 (0.27-1.11) | 0.095 |  | 0.66 (0.31-1.39) | 0.27 |
| Smoking (yes vs. no) | 2.00 (0.93-4.28) | 0.076 |  | 1.25 (0.56-2.81) | 0.58 |
| AJCC stage (III vs. I&II) | 2.24 (1.21-4.15) | **0.011** |  | 2.71 (1.40-5.22) | **0.003** |
| Bold values indicate significant P values (<0.05). CI = confidence interval. | | | | | |

**Table S5. Univariable and multivariable Cox regression analyses of BraMARS and clinical factors for brain metastasis-free survival**

| **Variables** | Univariable analysis |  |  | Multivariable analysis |  |
| --- | --- | --- | --- | --- | --- |
|  | Hazard ratio (95% CI) | *P* value |  | Hazard ratio (95% CI) | *P* value |
| **CHCAMS cohort** |  |  |  |  |  |
| BraMARS (high risk vs. low risk) | 9.82 (2.23-43.29) | **0.0026** |  | 8.77 (1.97-38.99) | **0.0043** |
| Age | 1.05 (1.00-1.11) | 0.067 |  | 1.08 (1.02-1.14) | **0.0087** |
| Gender (female vs. male) | 0.28 (0.06-1.25) | 0.096 |  | 0.27 (0.05-1.46) | 0.13 |
| Smoking (yes vs. no) | 1.85 (0.60-5.73) | 0.29 |  | 1.13 (0.30-4.17) | 0.86 |
| AJCC stage (III vs. I&II) | 5.71 (1.84-17.76) | **0.0026** |  | 7.42 (2.20-25.02) | **0.0012** |
| **HMUCH cohort** |  |  |  |  |  |
| BraMARS (high risk vs. low risk) | 4.31 (1.45-12.87) | **0.0088** |  | 4.77 (1.53-14.87) | **0.0071** |
| Age | 1.06 (1.00-1.13) | 0.062 |  | 1.08 (1.01-1.15) | **0.026** |
| Gender (female vs. male) | 0.77 (0.30-1.98) | 0.58 |  | 0.94 (0.35-2.54) | 0.9 |
| Smoking (yes vs. no) | 1.42 (0.52-3.90) | 0.49 |  | 0.85 (0.29-2.50) | 0.77 |
| AJCC stage (III vs. I&II) | 1.78 (0.69-4.59) | 0.23 |  | 2.62 (0.94-7.29) | 0.065 |
| Bold values indicate significant P values (<0.05). CI = confidence interval. | | | | | |
